# The Effect of Aging on Artificial Saliva at Different pH Values on the Color Stability of New Generation Denture Base Materials

**DOI:** 10.7759/cureus.55804

**Published:** 2024-03-08

**Authors:** Ezgi Arslan, Esra Nur Avukat, Canan Akay

**Affiliations:** 1 Department of Prosthodontics, Eskişehir Osmangazi University, Eskişehir, TUR

**Keywords:** graphene reinforced pmma, heat cure acrylic resin, artificial saliva, color stability, cad/cam pmma

## Abstract

Statement of problem: New-generation denture base materials are used successfully in denture fabrication; however, the effect of saliva pH change on the color stability of materials is unknown.

Purpose: The purpose of this in vitro study is to evaluate the color stability of new-generation denture base materials after immersion in artificial saliva with different pH values (3,7,14).

Material and methods: Disc-shaped samples (Ø 10 mm x 2 mm) were prepared from three different denture base materials (1 pre-polymerized polymethylmethacrylate [PMMA], 1 graphene-reinforced PMMA, and heat-cure polymethyl methacrylate resin) (n=10). After polishing, color coordinates were measured using a PCE-CSM 5 colorimeter programmed in the CIE system (L* a* b*). The samples were kept in artificial saliva at different pH values and 37°C for 21 days. At the end of 21 days, color coordinates were measured again. The suitability of the measurements for a normal distribution was examined with the Kolmogro-Smirnov test. Whether color measurements obtained at different pH levels differed according to groups was examined with the Kruskal-Wallis test. The correlation between the CIEDE2000 and CIELab color difference formulas was examined by correlation analysis.

Results: The highest color difference occurred in heat-cure samples at pH 3 (p<0.001). The color difference at different pH values was least observed in pre-polymerized PMMA samples. Significant color differences occurred in the graphene-reinforced pre-polymerized PMMA group at pH 7 (p<0.001).

Conclusions: It was observed that color differences occurred in all groups. Dentures made of new-generation CAD/CAM PMMA, which are less exposed to color differences, can be recommended for elderly patients with systemic diseases who are frequently exposed to pH changes in the oral cavity.

Clinical implications: Color differences on denture surfaces over time negatively affect aesthetics. Since pH changes cause changes on the prosthesis surface, it may be recommended for these patients to fabricate dentures from new-generation CAD/CAM PMMA resins, which are less deformable.

## Introduction

The conventional polymethylmethacrylate (PMMA) material used in the fabrication of complete dentures has acceptable mechanical and physical properties; however, it also has many disadvantages arising from the fabrication process. In recent years, complete dentures have been fabricated using computer-aided design and computer-aided manufacturing (CAD/CAM) technologies [1]. Since CAD/CAM blocks are fabricated under high temperature and pressure, the material has been reported to have more advanced properties than conventionally fabricated PMMA material. In addition, CAD/CAM PMMA has a lower residual monomer content compared to the denture base fabricated by conventional methods [2].

Graphene, known as the thinnest material in the universe, is frequently used in dentistry to improve mechanical properties. It is seen that water absorption rates decrease depending on the amount of graphene added to the PMMA material. It is thought that long-term color stability will be maintained by adding graphene to denture base materials that are constantly exposed to saliva in the mouth [3].

The dentures, which are in the mouth for most of the day, are in constant contact with saliva. In the contact of the polymer with saliva, some liquid absorption and release of monomers occur. The affinity of PMMA material for water absorption is independent of color pigments and dyes, and discoloration occurs with the absorbed water [4]. The degradation of acrylic resin material is explained in two ways: enzymatic reactions of saliva and hydrolysis of resin-containing materials. In addition, the constant acid-base balance of saliva pH, depending on the foods consumed during the day, also deteriorates the surface properties of PMMA material. Acidic environmental conditions can deteriorate the hardness of acrylic resin and increase the release of residual monomers [5]. Considering all these reasons, it is very important to choose a denture base material that can best withstand intraoral environmental conditions, chewing forces, and nutritional differences.

Saliva and its components are of great importance for the protection and maintenance of oral health. However, changes in the quantity and quality of saliva occur with aging. Approximately half of all adults have been reported to have symptoms of gastroesophageal reflux disease (GERD) at some point in their lives [4]. Diseases such as GERD increase the acidity of the saliva. In addition, systemic and autoimmune diseases such as diabetes, obstructive sleep apnea, and Sjögren's syndrome also cause a decrease in the amount of saliva. Many drug groups, such as antihypertensives, antihistamines, hypnotics, diuretics, and muscle relaxants, can also affect the function of the salivary glands, causing hyposalivation and a shift in salivary pH to acid. The alkalinity of saliva also increases with dysfunctions such as certain foodstuffs consumed, enzyme and secretion production of the pancreas, and liver problems. Although the disruptions of the pH balance to which the oral environment is exposed by the foods consumed can be limited by diet, saliva pH changes caused by the medications and diseases used by the patients cannot be prevented [6,7].

To ensure the long service life of a denture, it is necessary to pay attention to its cleaning and maintenance. There are many different brands of denture-cleaning products on the market. The common feature of these denture cleaners is that they contain a disinfectant with a high alkaline value, such as sodium hypochlorite (NaOCl). Although denture cleaners help to clean the denture, in the long term, the alkaline environment causes deterioration of the surface properties of the PMMA material and increased roughness. In addition, similar to acidic pH values, alkaline environments cause the hardness value of acrylic resins to decrease and color stability to deteriorate [8].

The color difference of the dental material can be evaluated through the thresholds of detectability and acceptability. CIELab and CIEDE2000 color difference formulas are widely used in dentistry to evaluate color differences. The CIEDE2000 color difference formula was developed to eliminate the shortcomings of the CIELAB (L*a*b) color difference formula. The CIEDE2000 color difference formula was found to be more appropriate and accurately compatible with its acceptability and perceptibility. In the study, the detectability threshold of acrylic resins was reported as 1.72, and the acceptability threshold was reported as 4.08, according to the CIEDE2000 formula [9].

One of the critical factors affecting the long-term success of dentures is the denture color difference. The aesthetics are affected by the denture color difference, causing denture renewal. Many studies have been conducted to evaluate the color stability of new-generation denture base materials by soaking them in pigment-containing liquids such as coffee, cola, and red wine. In line with the data from these studies, it has been observed that the materials change color at different rates [10,11]. However, studies evaluating the effect of pH values on color stability are limited.

The aim of this in vitro study is to evaluate the effect of changes in saliva pH due to different reasons on the color stability of new-generation denture base materials. In addition, since the number of studies published with the CIEDE2000 color difference formula is low, it was aimed to compare CIELab and CIEDE2000 color formulas and determine which one reflects the color difference better.

## Materials and methods

Material

**Figure 1:**
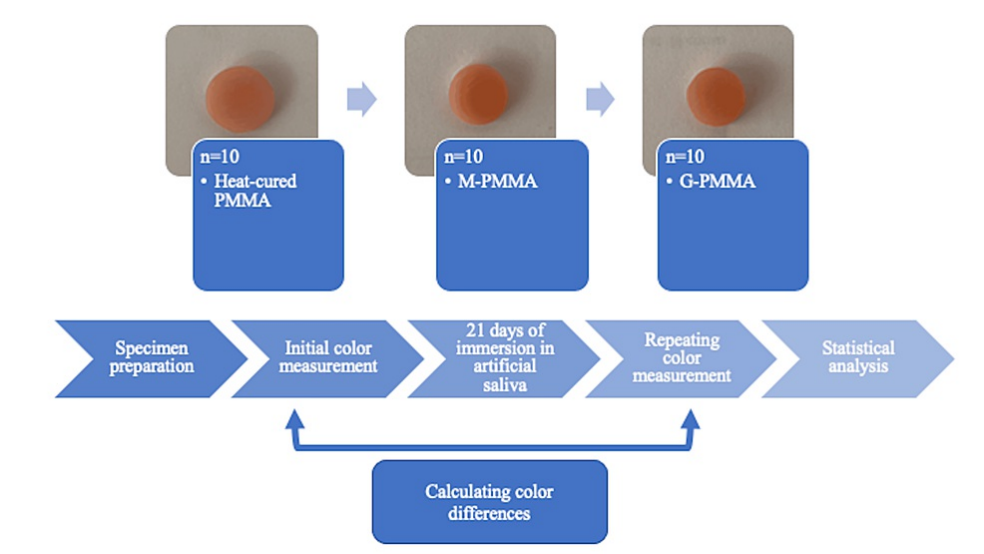
Study design PMMA: polymethylmethacrylate; M-PMMA: Merz polymethylmethacrylate; G-PMMA: nanographene-reinforced polymethylmethacrylate

A conventional heat-cure PMMA and two different brands of prepolymerized CAD/CAM denture base materials (1 pre-polymerized PMMA and 1 graphene-reinforced PMMA) were used in the study. The materials and their contents are shown in Table 1, and the design diagram of the study is shown in Figure 1. 

**Table 1:** Materials used in the study and their contents PMMA: polymethylmethacrylate; M-PMMA: Merz polymethylmethacrylate; G-PMMA: nanographene-reinforced polymethylmethacrylate

Material	Composition	Type	Code	Chemical Composition
Paladent PMMA	Heat-polymerized PMMA	Heat-cure PMMA	Heat- cure PMMA	Polymethylmethacryate, ethylmethacrytlate, methyl methacrylate, N-octyl methacrylate, glycol dimethacrylate, dimethyltouludine
Pink M-PM PMMA	Prepolymerized PMMA	Subtractively manufactured denture base resin	M-PMMA	Polymethylmethacrylate and cross-linked polymers based onmethacrylic acid esters, colorants, residual peroxide asdibenzoyl peroxide, methylmethacrylate (MMA) may be contained as residual monomer up to max 1%
Graphene Reinforced PMMA	Prepolymerized Graphene Reinforced PMMA	Subtractively manufacturednanographene-reinforced denture base resin	G-PMMA	Not disclosed

Specimen preparation

Heat-cure PMMA samples were processed by conventional flasking and pressure pack techniques. Disc-shaped wax samples (Æ10x2 mm) were placed in flasks, and the wax was removed by keeping them in boiling water for 10 minutes [12]. Heat-cure PMMA material (Paladent 20, Heraus Kulzer GmBH&Co., Germany) was mixed at 23.4 g/10 mL powder/liquid according to the manufacturer's instructions and mixed up at room temperature (23 ± 2°C) for 60 seconds. After 5 minutes, it was poured into the containers with the help of a spatula until it reached a dough consistency. For polymerization, the flasks were placed in room-temperature water and heated to 74°C. After standing at this temperature for 30 minutes, it was heated to 100°C, and the flasks were allowed to cool in a water bath to room temperature for 30 minutes. Samples whose polymerization was complete were removed from the flasks. Excess parts of the samples were removed with hand tools and tungsten carbide burs.

Pre-polymerized PMMA disk samples were cut into 2 mm-thick cylindrical samples with a diameter of 10 mm obtained from the PMMA block using a diamond blade (Buehler IsoMet Diamond Wafering Blad, Series 15 LC, No. 11-4276, Buehler, USA) in the cutting device (IsoMet 1000, Censico International Pvt). All surfaces of the samples were polished in the order of 1000-1200-2000 grit silicon carbide paper (3M wet abrasive sheet, Minnesota, USA) to obtain standardized smooth surfaces [13]. Heat-cure PMMA samples were soaked in distilled water for 24 hours to reduce the amount of residual monomer. 

Color coordinate measurements

Samples were visually evaluated before color coordinate measurements to check for deformation or irregularity. Baseline color coordinate measurements of the samples with completed surface treatments were carried out using a PCE-CSM 6 colorimeter (PCE instruments, Meschede, Germany) device programmed in the CIE system (L*, a*, b*). According to the CIE (Commission International de I'Eclairage) L* a* b* Color System, the L* (light) coordinate, which forms the vertical axis (y), indicates the brightness of the object. The L* value varies between 0 and 100. The changing L* value “0” represents black, and the value “100” represents white. Negative (-) values of the a* coordinate, which forms the X-axis, indicate color saturation between green and red, and positive values (+) indicate color saturation between purple and red. The b* coordinate that forms the Z axis means that (-) values indicate blue color saturation, and (+) values indicate yellow color saturation. The intersection of these three coordinates gives the value of that color. Before measurement, the device was calibrated according to the manufacturer's instructions*. Measurements were made under D65 illumination (corresponding to average daily light).

Artificial saliva preparation

Artificial saliva with different pH values was then prepared, and the samples were subjected to aging for 21 days [14]. Artificial saliva contains 4.1 mM KH2PO4, 4.0 mM Na2HPO4, 24.8 mM KHCO3, 16.5 mM NaCl, and 0.25 mM CaCl2. Artificial saliva was adjusted to 1 milliliter per sample, then pH 3, pH 7, and pH 14 were obtained by adding NaOH or HCl. The artificial saliva was sterilized by filtration before use [14]. The samples were placed in the sample container with all surfaces exposed to saliva and kept for 21 days at 37°C in the presence of 100% humidity [15].

Color coordinate measurements after aging

At the end of 21 days, the samples were removed and disinfected with 70% alcohol. Then, color coordinate measurements of the samples were repeated. L*, a*, and b* values of the samples were determined before the procedure and after they were kept in artificial saliva with different pH values for 21 days.

For an objective evaluation of color differences, the CIELab color difference formula (ΔE*) and CIEDE2000 (ΔE00) were used with the color difference formula as follows: 

ΔE*= [(ΔL*)2+ (Δa*)2+(Δb*)2)]1/2,

ΔE00= [(DL’/kLSL)2 + (DC’/kCSC)2+ (DH’/kHSH)2+ Rt(DC’/kCSC)2(DH’/kHSH)2]1/2.

For the CIEDE2000 color difference formula, the parametric factors of KL, KC, and KH were set to 1 [16]. 

Statistical analysis

Whether the measurements were normally distributed or not was analyzed with the Kolmogorov-Smirnov test. Heat-cure: the color values obtained at different pH levels in the Merz and graphene-reinforced groups are determined by the Friedman and Kruskal-Wallis test, in which the color coordinate measurements obtained at different pH levels do not differ according to the groups. It is distinguished by a gap analysis of the relationship between CIEDE2000 and CIELab measurements. Analyses were made with IBM Corp. Released 2011. IBM SPSS Statistics for Windows, Version 20.0. Armonk, NY: IBM Corp. at a 95% confidence level.

## Results

**Figure 2:**
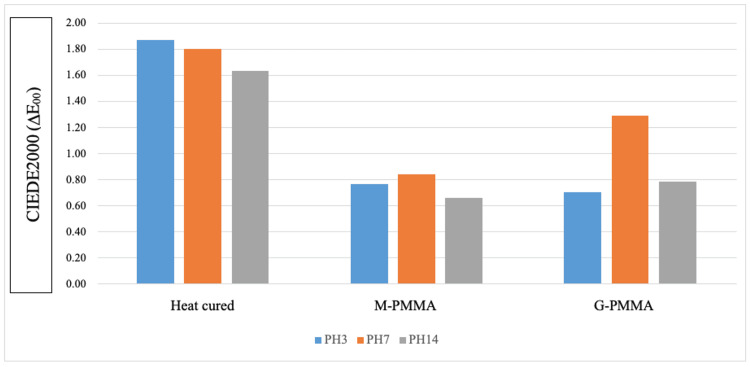
Color differences at different pH values according to CIEDE2000 data PMMA: polymethylmethacrylate; M-PMMA: Merz polymethylmethacrylate; G-PMMA: nanographene-reinforced polymethylmethacrylate

Color differences due to artificial saliva aging for different PMMA materials are presented in Table 2 and Figure 2. When the differences between groups and pH in CIEDE2000 data are examined, there is no significant difference between different pH values in the heat-cure and merz groups. The pH 7 measurement color difference in the graphene-reinforced group is significantly higher than the pH 3 group.

**Table 2:** Color differences between different pH values according to CIEDE2000 data a-b: There is no difference between groups with the same letter within each column; 1-2: There is no difference between groups with the same number in each row. PMMA: polymethylmethacrylate; G-PMMA: nanographene-reinforced polymethylmethacrylate; M-PMMA: Merz polymethylmethacrylate; SD: standard deviation (p<0.05).

CIEDE2000	pH 3	pH 7	pH 14	p
	Mean±SD	Mean±SD	Mean±SD	
Heat-cure PMMA	1,874 ^(a) ^±0,545	1,802^(a) ^±1,094	1,634^(a) ^±1,044	0,273
M-PMMA	0,767 ^(b)^ ±0,376	0,842^(b)^±0,393	0,662^(b) ^±0,337	0,497
G-PMMA	0,704 ^(b)(1)^ ±0,215	1,289^(c)(2) ^±0,419	0,786^(b)(3) ^±0,319	0,006
p	<0,001	<0,001	<0,001	

When the difference between the groups at different pH levels was examined, a significant difference was observed between the groups in all pH groups. In pH 3 measurements, the heat-cure group measurement average is significantly higher than the Merz and graphene-reinforced groups. The average of the heat-cure group in pH 7 measurement is significantly higher than the Merz group.

In the pH 14 group, the heat-cure group measurement average is significantly higher than in the Merz and graphene-reinforced groups.

At pH 3, the least average color difference occurred in pre-polymerized graphene-reinforced groups; an average color difference was observed in the slightly pre-polymerized Merz group at pH 7 and 14. Furthermore, a significant difference in color occurred in the graphene-reinforced pH 7 group. The color difference in pre-polymerized PMMA samples is slight but not significant (p<0.05).

**Figure 3:**
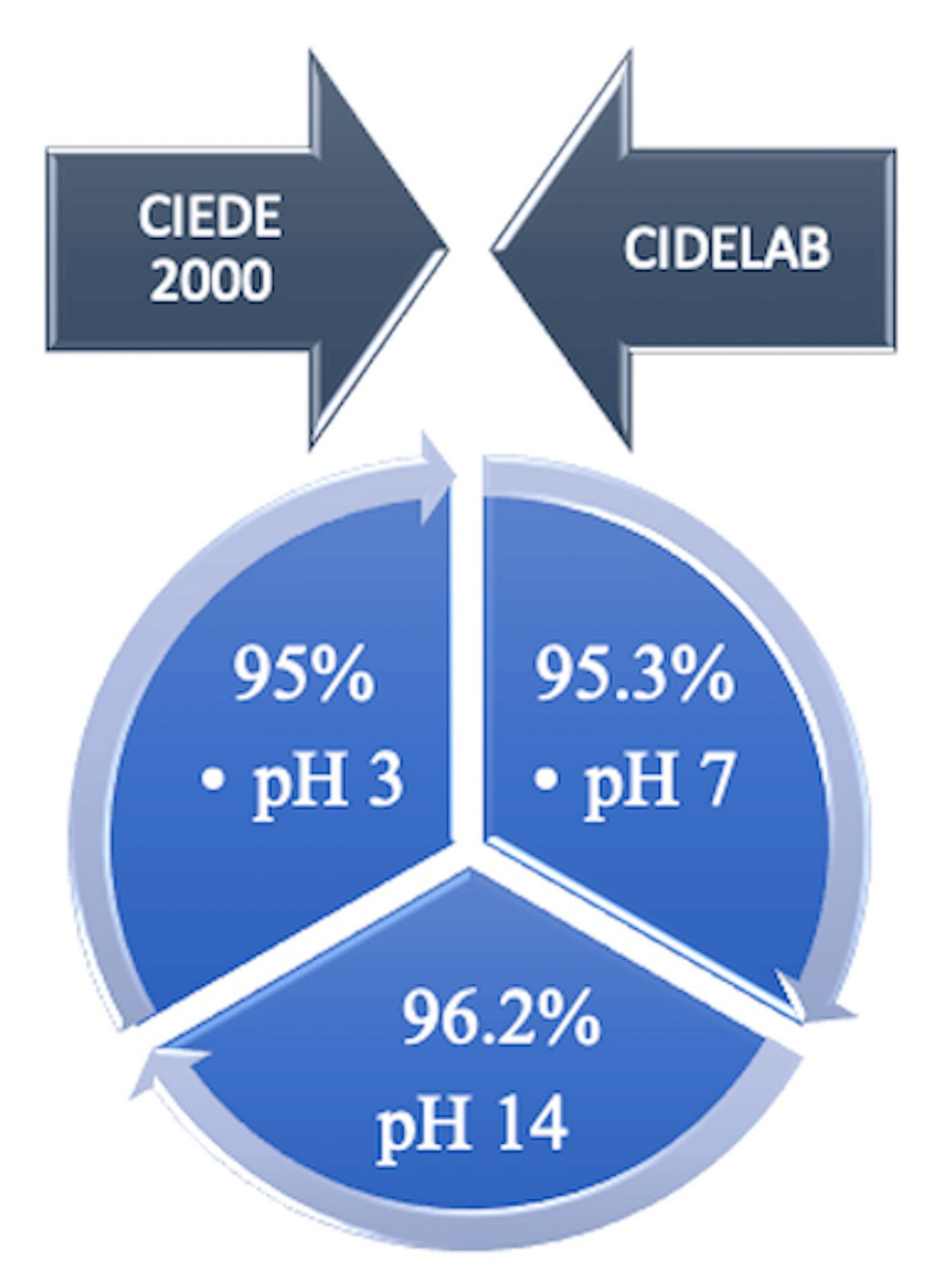
Positive correlation between CIEDE2000 and CIELab color difference formulas

The correlation solution between CIEDE2000 and CIELab measurements shows a significant positive correlation at pH 3 for 95%, pH 7 for 95.3%, and pH 14 for 96.2% (Table 3 and Figure 3). 

**Table 3:** Correlation solution between CIEDE2000 and CIELab measurements **p<0.001

		pH 3_lab	pH 7_lab	pH 14_lab
pH 3	r	.950^**^	.048	.510^**^
	p	.000	.801	.004
pH 7	r	-.026	.953^**^	.142
	p	.892	.000	.456
pH 14	r	.529^**^	.163	.962^**^
	p	.003	.390	.000

## Discussion

In this study, the effect of saliva pH, which is affected by the patient's systemic conditions, food, and beverages consumed during the day, on the color stability of denture base materials was examined. Three different denture base materials were used in the study. Once the surface preparations of the samples were completed, baseline color coordinate measurements were made with a colorimeter device. Then, the samples were kept in artificial saliva at different pH values (3, 7, and 14) for 21 days. At the end of 21 days, the color coordinate measurements of the samples were repeated. The ΔE values of the samples were calculated according to the CIEDE2000 and CIELab color coordinate measurement systems. The main conclusion of this in vitro study is that aging different types of PMMA denture base materials in artificial saliva causes color differences. According to the results, the heat-cure PMMA group has significantly the highest ΔE values. In line with the findings, the hypothesis that the color stability of PMMA materials aged in artificial saliva at different pH values will be affected is accepted. 

The pH value of the oral environment can affect the surface properties of denture base materials. Kazazoğlu et al. examined the surface properties and hardness changes of denture base materials obtained using different production methods after being immersed in gastric acid with a pH between 1 and 1.5. When all acrylic resin groups were exposed to stomach acid, surface roughness increased, and hardness values decreased. As the exposure time increased with the acidic pH value, the denture base materials fabricated with CAD/CAM showed better resistance to acidic pH values [17]. Similarly, in the study conducted, the highest color change at acidic pH values was observed in the heat-cure PMMA material.

In another study by Tieh et al., the effects of various liquids (artificial saliva, coffee, wine, and denture cleaner) on conventional, milled, and 3D-printed denture teeth were examined. While color differences occur in samples in pigment-containing environments such as coffee and wine, they have also been reported in samples stored in artificial saliva and denture cleaner. Denture cleaners were reported to cause the highest optical and mechanical effects among all groups. It was reported that this may be due to the material absorbing water or the alkaline environment created by the ingredients of the denture cleaner deteriorating the surface properties of the material [18]. Although there were mediums that did not contain color pigments in the study, color differences occurred in all acrylic resins. This color difference occurred mostly in the heat-cure PMMA material.

The clinical performance of dental materials depends on their mechanical properties, such as long-term success and wear resistance. In a study, the mechanical properties, water absorption, and morphological properties of a PMMA resin added with graphene-silver nanoparticles (Gr-Ag) were evaluated. As a result of the study, it was seen that the addition of Gr-Ag could not only improve the mechanical properties of the material but also reduce the effects of deterioration due to liquid absorption in the long term by reducing water absorption [3]. In the study, it was observed that graphene-reinforced PMMA material-maintained color stability better compared to heat-cure PMMA material. However, graphene-reinforced PMMA showed more color differences in the environment at pH 7 than in the environment at alkaline and acidic pH values.

**Figure 4:**
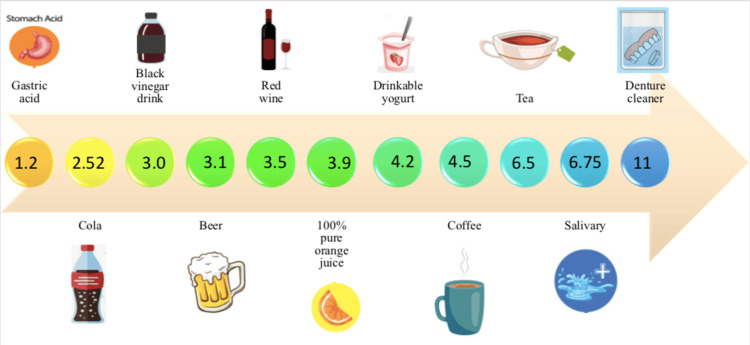
Some liquids and pH values may cause denture discoloration The figure is not taken from a work, it is original and made by me.

Although color is initially thought of as pigments or dyes added to the surface or mixed within the material, color is the effect of light waves passing through the object and reflecting back. Since surface distortion affects the reflection of light, it can also change the perceptibility of color. Dentures are in contact with many different foods and drinks during the day, which can cause their surface deteriorate (Figure 4) [19-22]. 

Patients can clean their dentures using mechanical and chemical methods. Although denture cleaning is mostly done with mechanical methods such as brushing, chemical methods are a good alternative for patients with weakened motor functions. Disinfection processes cause some changes in the denture surfaces. Macédo et al. evaluated the effect of cinnamaldehyde, which has an antifungal effect and is used in cleaning dentures, on the denture base material after five years of use. In the study, 80 PMMA samples polymerized with microwave heat were obtained, and then these samples were immersed in four different liquids (TWetap water [control], SH e 0.5% sodium hypochlorite, PXealkaline peroxide, and CAecinnamaldehyde [27 mg/mL]). At the end of the process, color differences were measured with a spectrophotometer and surface roughness with a noncontact optical profilometer. Although it may cause changes on the PMMA surface after the use of denture disinfectants with high pH values, it has been reported that it is suitable for clinical use. However, values higher than those used in the study may affect the result of the study and increase surface distortion. Water absorption of the material after denture cleaners, release of residual monomer, and formation of microcracks in the material change the surface properties of the material, negatively affecting the color [23]. In this study, artificial saliva with an alkaline pH value, similar to alkaline denture cleaners, also causes color differences. However, for heat-cure PMMA and Merz-group prepolymerized PMMA material, the environment at pH 3 and pH 7 caused more color differences in the materials.

Some of the factors that cause the color stability of denture base materials to deteriorate include exposure to disinfectant substances that can change the surface over time and exposure to oral fluids. In a study evaluating the effect of denture cleaners on the color stability and surface roughness of PMMA denture bases fabricated with different techniques, it was observed that denture cleaners with alkaline pH, especially NaClO, affected the surface roughness and color stability of PMMA. Among the PMMA acrylic denture bases fabricated by three different methods, the highest surface roughness was found in the thermosetting PMMA material group [24].

It is aimed at obtaining clearer results than the visual color scale of measurements made using different color devices. While the eye perceives the changes resulting from the difference in tone more clearly, it has difficulty perceiving the color differences resulting from the difference in brightness. Although the CIELab color difference formula has been used for many years, new color difference formulas have been developed due to its shortcomings. The newest and most recommended one is CIE2000 or CIEDE2000. It has been reported that the current color difference formula can detect color differences more conveniently and accurately [25]. It is considered suitable for clinical use if the color difference is <3.7 [26]. Although the highest ΔE was found to be 3.9284 in this study, when the samples were considered in general, the ΔE value was found to be <3.7. Although artificial saliva at different pH values causes color differences, it is suitable for clinical use.

The limitation of this study is that a single environment was evaluated at three different pH values. Color stability could also be evaluated by including solutions containing different color pigments and dyes. Besides, the addition of PMMA material fabricated by 3D printing, one of the new generation denture base materials, to this study may help with denture base material problems for which there is not yet sufficient information in the literature. More research is needed to evaluate the effects of saliva pH on CAD-CAM materials.

## Conclusions

According to the results of the study, in which the color differences created by the effect of artificial saliva at different pH values on heat-cure and new-generation CAD/CAM PMMA denture base materials were evaluated, a change in the optical properties of all samples occurred as a result of aging with pH. The highest color difference values were observed in heat-cure PMMA samples. The merz PMMA material group had the lowest color difference values after being immersed in artificial saliva. In patients where saliva pH change cannot be prevented, dentures fabricated with the new-generation CAD/CAM PMMA material will better maintain color stability and provide long-lasting dentures. It has been observed that there is a positive correlation between the CIEDE2000 and CIELab color difference formulas used during color calculations.
